# Diurnal rhythmicity of wearable device-measured wrist temperature predicts future disease incidence in the UK Biobank

**DOI:** 10.21203/rs.3.rs-2535978/v1

**Published:** 2023-02-15

**Authors:** Carsten Skarke, Thomas Brooks, Nicholas Lahens, Gregory Grant, Yvette Sheline, Garret FitzGerald

**Affiliations:** University of Pennsylvania; University of Pennsylvania; Institute of Translational Medicine and Therapeutics (ITMAT), University of Pennsylvania; Department of Genetics, University of Pennsylvania Perelman School of Medicine; University of Pennsylvania Perelman School of Medicine; University of Pennsylvania

## Abstract

Many chronic disease symptomatologies involve desynchronized sleep-wake cycles, indicative of disrupted biorhythms. This can be interrogated using body temperature rhythms, which are well-established biomarkers for circadian clock function. Here, we investigated the association of wrist temperature amplitudes with a future onset of disease in the UK Biobank one year after actigraphy. Among 425 disease conditions (range *n* = 200–6,728) compared to controls (range *n* = 62,107 − 91,134), a total of 73 (36.5%) disease phenotypes were significantly associated with decreased amplitudes of wrist temperature (Benjamini-Hochberg FDR q < 0.05) and 26 (13%) PheCODEs passed a more stringent significance level (Bonferroni-correction α < 0.05). Here, for example, a two-standard deviation (1.8° Celsius) lower wrist temperature amplitude corresponded to hazard ratios of 1.91 (1.58–2.31 95% CI) for NAFLD, 1.69 (1.53–1.88) for type 2 diabetes, 1.25 (1.14–1.37) for renal failure, 1.23 (1.17–1.3) for hypertension, and 1.22 (1.11–1.33) for pneumonia. A comprehensive phenome-wide atlas of the identified mappings has been made available at http://bioinf.itmat.upenn.edu/biorhythm_atlas/. These findings strongly suggest peripheral thermoregulation as a digital biomarker.

## Introduction

The benefits of regular physical activity and sufficient sleep are pillars of public health^1,2^. Efforts to assess this linkage objectively at the population level have utilized accelerometry in a phenome-wide scan to associate physical inactivity with a broad range of chronic diseases in the UK Biobank (UKBB)^3,4^ and the “All of Us” Research Program^5^. The role biorhythms play in predicting disease incidence is less well understood^6^. Though a few disease-specific studies, limited to patients with mood disorders,^7,8^ have measured the degree of disrupted rest-activity cycles from accelerometry in the UKBB, a comprehensive phenome-wide approach has not yet been established.

Temperature rhythms are well-established biomarkers for circadian clock function^9^. Peripheral wrist temperature oscillations serve as a proxy for endogenous circadian function - through the thermoregulatory coupling of core and periphery - and run inverse to the core clock^10,11^. These traces retain an endogenous sinusoidal component under conditions of constant temperature and light with standardized food intake^11^ and are therefore suitable to estimate circadian entrainment, comparable to melatonin or core body temperature, as marker rhythms^12,13^. Shift work reduces the amplitude of temperature rhythms^14^ and disruption of temperature rhythms has been strongly associated with metabolic syndrome and diabetes^15^, as well as sleep-disordered breathing^16^.

To build on this and to explore the relationship between wrist temperature amplitudes and the future onset of diseases, we utilized temperature data collected through an embedded sensor in the actigraphy device that was used for the UKBB study,^17,18^.

## Results

### Wrist Temperature Readings are Concordant with a Biorhythm Signal

**Figure 1:**
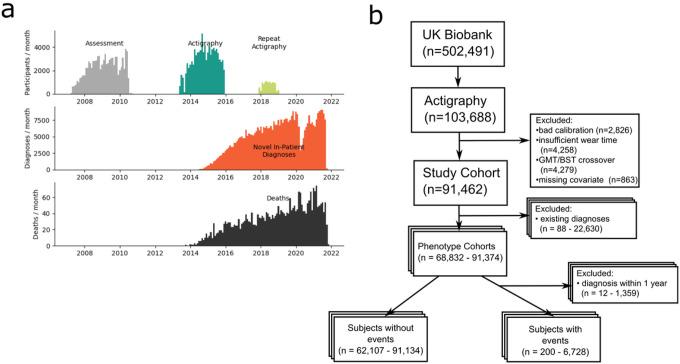
Study Design The UK Biobank actigraphy study yielded 91,462 individuals with high-quality actigraphy health measurements and complete covariate data. A total of 451,994 distinct patient-diagnoses (4.9 per participant) and 3,061 deaths (3.3%) were recorded among these participants as per data downloaded on February 23, 2022. (a) Timeline of data collection by year. Participants had a mean of 5.7 years between covariate assessment and actigraphy collection and a mean of 5.9 years of follow-up starting one year after actigraphy. (b) Flow diagram of participants. For each phenotype, subjects were excluded from analysis if they had prior diagnosis of the PheCODE or of any of the PheCODEs defined as exclusion criteria by the PheCODE map. Diagnoses within one year of actigraphy were also excluded, due to the likelihood of disease onset occurring before diagnosis.

**Figure 2:**
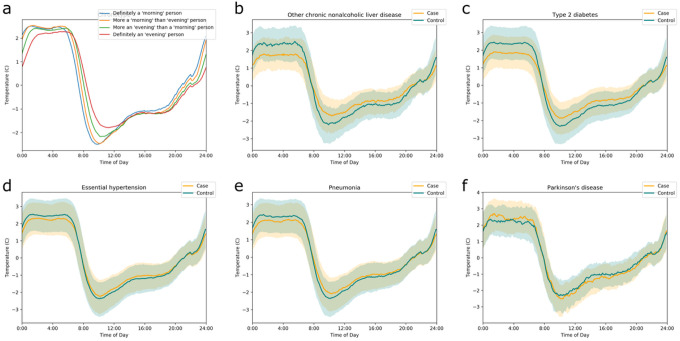
Wrist temperature traces Wrist temperature traces by (a) chronotype (morningness/eveningness), and by case-status in pairs matched by age and sex for (b) NAFLD, (c) type 2 diabetes, (d) hypertension, (e) pneumonia, and (f) Parkinson’s disease. Please note that the temperature curve for Parkinson’s disease in Panel (f) separates from the controls but with opposite directionality compared to the disease conditions displayed in panels (b-e). The 25^th^ to 75^th^ percentiles of the population are displayed in shaded regions (controls in blue, cases in yellow and overlap in grayish green). Wrist temperature is normalized so that each individual’s daily average is 0.

The UKBB collected 7 days of actigraphy from 103,688 participants (Fig. 1)^19^, with 91,462 participants passing quality control and having all covariates. The wrist actigraph device housed a temperature sensor near the skin. Though its primary purpose was related to accelerometer calibration^17^, we found that the peripheral skin temperature signal (Figure S1) was strong enough to produce a phenotype characteristic of a biological origin. This wrist temperature signal was clearly discernable despite it being modulated (“masked”) by sleep, ambient temperature conditions, food intake and other confounders. The shape of the UKBB wrist temperature curves matched previously reported curves for peripheral skin measured at the wrist^11^. In the wild, wrist temperature readings characteristically increase with sleep onset, plateau during sleep and then drop suddenly upon awakening - followed by a second smaller peak in the afternoon^11^. The shape depicted in Fig. 2a is consistent with this phenotype. The plateau of maximum wrist temperatures occurs during night hours (Fig. 2a, Figure S2). This shape has been described as running phase-advanced or inverse (anti-phase) to the core body temperature^20^. The relationship of cooling down the body core temperature by peripheral vasodilation resulting in heat loss has been proposed as a thermoregulatory mechanism to induce sleep^21^. The peak-to-trough variation in the observed wrist temperature in Fig. 2 matches the 6°C differential between maximum (36.1 ± 0.5°C) and minimum (30.4 ± 1.7°C) diurnal temperature fluctuations reported for a cohort of 103 healthy volunteers^22^. The similarity in these data underscores the confidence in the methodology. The wrist temperature curves are right shifted for the evening chronotype compared to participants reporting a morning preference (Fig. 2a). This corresponds well with the 2–3 hour delay in circadian phase observed between morning and evening chronotypes^23^. To underscore the value of wrist temperature as a potential biomarker, we refer to a study in 13 healthy volunteers showing that phase determined by wrist temperature readings correlated strongly (R = 0.756) with the circadian phase assessed by dim light melatonin onset (DLMO), which is the gold standard in the eld^13^.

### Biorhythms in Wrist Temperature Predict Future Diagnoses

**Figure 3:**
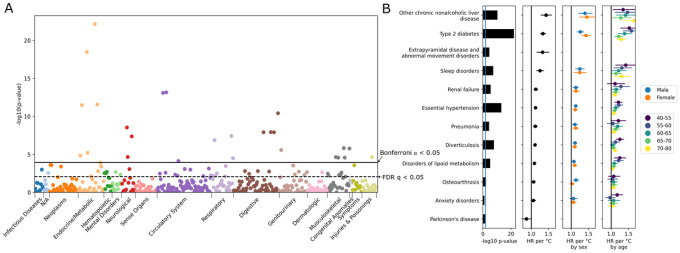
Diurnal Rhythmicity Predict Diagnoses To test whether diurnal rhythmicity predicts future disease, a Cox proportional hazards model was performed for each phenotype (PheCODE), to test for effects from the wrist temperature amplitude. Individuals with diagnoses prior to the actigraphy measurement were excluded as well as those with a new diagnosis code within the first year following actigraphy. (a) Manhattan plot-style display of phenome-wide results. In many phenotypes, weaker wrist temperature rhythms predict future onset of disease. The solid line shows Bonferroni-correction significant at α ≤0.05 and dashed line shows Benjamini-Hochberg FDR at 0.05. (b) A selection of significant phenotypes shown in detail. Left: significance of the overall effect size (irrespective of age and sex). Right: three panels, effect sizes of the overall model, by sex model, and by-age model. Effect size measured as the hazard ratio (HR) per 1°C decrease in the wrist temperature amplitude, and lines denote the 95% CI. Extrapyramidal disease and Parkinson’s disease had insufficient cases to run by age and sex. Anxiety disorders and Parkinson’s disease are slightly above the *q*<0.05 threshold, at *q*=0.20 and 0.11, respectively. No phenotypes displayed significant differences by sex or by age after correction for multiple testing (q>0.5 for all phenotypes).

Disease phenotypes were extracted from medical records and grouped according to the PheCODE Map^24,25^. To investigate how well wrist temperature amplitude predicts future diagnoses, Cox proportional hazards models were computed with each PheCODE diagnosis as an endpoint and the temperature amplitude as a predictor. To exclude participants with subclinical disease at the time of actigraphy, we limited enrollment to those participants whose first recorded diagnostic event was at least one year after their actigraphy assay. Of the 425 PheCODEs with at least 200 cases, a total of 73 (36.5%) reached significance at a Benjamini-Hochberg false discovery rate (FDR) of *q* < 0.05 (Fig. 3a, Table S 2). In each of eight categories (neoplasms, neurological, digestive, genitourinary, respiratory, musculoskeletal, circulatory system, and endocrine/metabolic conditions) five or more PheCODEs were significant at this level, highlighting the importance of interrogating across the human phenome. The more conservative Bonferroni family-wise error rate (FWER) of α < 0.05, (*p* < 0.00012, Fig. 3a), identified a total of 26 (13%) PheCODEs. Here, the most significant associations represent common chronic disease phenotypes which have been associated with circadian disruption^6,26^.

The following vignettes explore selected disease predictions in more detail. A two-standard deviation (SD) decrease (1.8°C) in wrist temperature amplitude (HR = 1.91 [1.58–2.31, 95% CI], *q* = 2.3×10^− 9^, n = 603 events) is associated with nearly double the rate of nonalcoholic fatty liver disease (NAFLD) disease. The most common billing codes for this PheCODE were steatosis (*n* = 533 for ICD10 K76.0) and cirrhosis (n = 79 for ICD10 K74.6). This corroborates the known loss in diurnal variance of distal skin temperature that has been observed in cohorts of patients with liver cirrhosis compared to controls^27^. Consequently, the distal-proximal temperature gradient indicative of heat dissipation is blunted, which contributes to the highly prevalent sleep disruptions reported for cirrhotic patients^28^, among other proposed mechanisms such as displaced melatonin secretion^29^. This raises the question of whether repeated weeklong wrist temperature readings, assessing diurnal variability in the thermoregulatory system, constitutes a valid digital biomarker to monitor disease progression.

Onset of diabetes has long been associated with circadian clock disruption^30^. The ensuing peripheral vascular disease and neuropathy impair thermoregulation specifically at night^31,32^. The resulting reduction in the distal temperature amplitude has been observed in diabetic patients before polyneuropathy manifested^31^, suggesting that altered peripheral temperature rhythms identify patients early at risk of disease progression. In the UKBB data, type 2 diabetes mellitus has an HR of 1.69 (1.53–1.88, 95% CI, *q* = 2.9×10^− 20^, n = 1,936 events).

Wrist temperature rhythms also had large predictive power in hypertension (HR = 1.23 [1.17–1.30), *q* = 8.4×10^− 12^, *n* = 6143) and in disorders of lipid metabolism (HR = 1.16 [1.09–1.24] *q* = 1.4×10^− 4^, *n* = 4,072), consistent with the link to metabolic syndrome^33^.

The 2 SD decrease in wrist temperature amplitude predicted a 67% increase in new onset of extrapyramidal disease and abnormal movement disorders (HR = 1.67 [1.32–2.11), *q* = 4.4×10^− 4^, *n* = 293). Diurnal variation in dopamine neurotransmission is well known^34^ with evidence for functional consequences. Drug-induced evocation of extrapyramidal symptoms, akathisia and dystonia, in patients with schizophrenia was more severe at night compared to the morning hours^35^. This relationship is likely modulated by the high degree of inter-individual variability in the dopamine D2 receptor density^36^, which, with its role in central thermoregulation, may lead to the high predictive power between wrist temperature and susceptibility to extrapyramidal disease.

Psychiatric disorders are commonly associated with altered circadian rhythms^37^. In the present study, anxiety disorder was one of the few mental PheCODEs with more than 200 cases to show a trend (HR = 1.11 [0.99–1.24), *q* = 0.20, *n* = 1,555) (Fig. 3b, Table 1).

Associations with the opposite directionality were observed in just a few PheCODEs. Decreased wrist temperature rhythms were associated with decreased future diagnoses for Parkinson’s disease (HR = 0.76 [0.59–0.97], *q* = 0.11, n = 295) (Fig. 2f), and with Raynaud’s syndrome (HR = 0.63 [0.51–0.79), *q* = 0.0011, *n* = 295). These are unexpected findings. However, both of these diseases are marked by impaired thermoregulation^38,39^, linked in Parkinson’s to alpha-synuclein pathology in the CNS^39^ and in Raynaud’s to sympathetic nervous system and α2C adrenoceptor dysregulation in the small blood vessels of the digits^40^. In Parkinson’s, the peripheral temperature trace is likely altered by episodic hyperhidrosis^38^ particularly in the dysautonomic subtype^41^. The accelerometry trace in Parkinson’s patients indicated lower diurnal variation and physical activity (Figure S3), an observation associated with elevated risk of disease^42^. Here, time-specific deep phenotyping studies, like those performed by the human chronobiome initiative^43^, are necessary to disentangle disease mechanisms.

### Sex- and Age-Dependent Differences in Wrist Temperature Biorhythms

Trends for sex-dependent differences emerged for several disease phenotypes. Hernias, degenerative joint diseases, reflux esophagitis and colorectal cancer showed a weak interaction between sex and wrist temperature amplitude (*p* < 0.05); however, to the extent that these are real, there was insufficient power to overcome correction for multiple testing (*q* > 0.5 for all PheCODEs).

Similarly, trends for age-dependent differences were present in age-related diseases, such as diabetes, cognitive disorders, cataract, neoplasms, heart valve disease and diverticulosis (*p* < 0.05, *q* > 0.5 for all PheCODEs).

### Blunted Rhythms in Wrist Temperature Associated with Higher All-Cause Mortality

Overall, subjects with lower amplitude had increased risk of all-cause mortality (*p* = 0.002, *n* = 3,061 deaths HR = 1.14 [1.04–1.24, 95% CI] for a 2 SD decrease in amplitude). Age, fitted as a linear relationship with the outcome, was controlled in that model. To confirm age-independence of this relationship, the association between decreased amplitude and mortality remained significant when age categories of under and over 65 years of age were analyzed separately (*p* = 0.037, HR = 1.10, *n* = 759 deaths for < 65 years of age compared to *p* = 0.018, HR = 1.06, *n* = 2302 deaths for > 65 years of age). Interestingly, mortality associations were not consistently significant among sub cohorts (see Supplemental Methods), possibly driven by lower death counts or covariates such as recruitment center.

### Temperature Biorhythm Atlas

**Figure 4:**
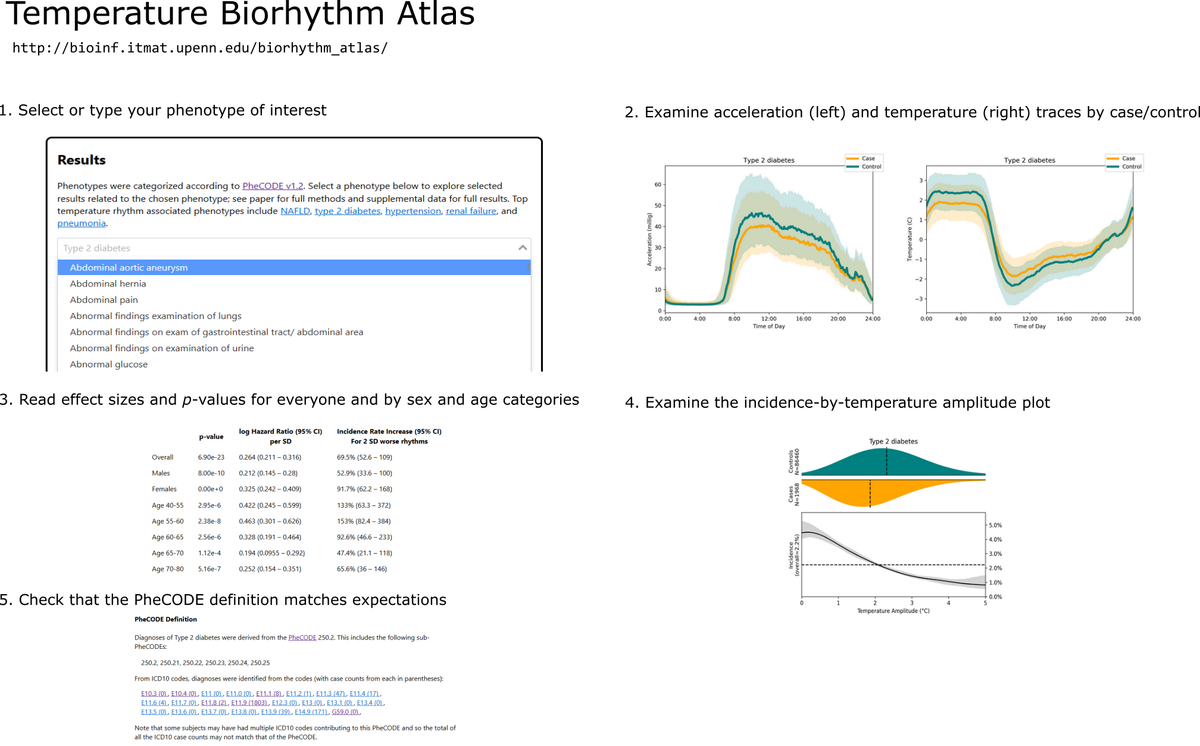
Temperature Biorhythm Atlas Guide We have made the results of this study easily explorable on a web-based atlas (http://bioinf.itmat.upenn.edu/biorhythm_atlas/). Users select a phenotype (PheCODE) of interest from a drop-down list. Then, they are presented with results pertaining to that phenotype. First, average traces by time-of-day are shown for matched case/control pairs, as in Figure 2. Next, effect sizes and statistical significance in a tabular format, broken down by sex and age (for phenotypes with sufficient case counts in each category). Then a plot of distributions of wrist temperature amplitudes in both the cases and controls, along with a plot of the incidence rate of the disease stratified by temperature amplitude rhythms. Non-constant incidence rates indicate an association of rhythm with disease. Lastly, details about the definition of cases and controls for the phenotype are given, including the specific ICD-10 codes (with case counts) used to identify cases, as well as the exclusion criterion. These allow investigators interested in a single phenotype to quickly assess it for connections to diurnal rhythm disruption.

To enable phenome-wide access to the results, we created a comprehensive temperature biorhythm atlas to visualize and quantify how the diurnal phenotypes of wrist temperature modulate the rate of diagnosis for a future disease of interest following the International Classification of Diseases 10th Revision (ICD-10) codes. Diurnal acceleration traces are provided as visual reference. This atlas (http://bioinf.itmat.upenn.edu/biorhythm_atlas/) serves as a resource for clinicians, researchers, and the public to consider temperature biorhythms as a potential digital biomarker for the future onset of diseases (Fig. 4).

## Discussion

We identified 73 out of 425 (36.5%) disease phenotypes across the human phenome that are predicted by decreased wrist temperature amplitudes; 26 of which (13%) were highly significant under more stringent criteria. By eliminating potentially undiagnosed mild or subclinical disease conditions during the monitoring period we decreased the risk of reverse causation where existence of an underlying disease condition might have dampened the temperature rhythms. Among the best predicted diseases were NAFLD, diabetes mellitus, hypertension, asthma, chronic airway obstruction, lipid disorders, chronic liver disease, renal failure, and pneumonia disorders. Most of these belong to the group of chronic conditions responsible for 90% of the $4.1 trillion spent annually on health care in the US^44^.

The strength of our phenome-wide approach is the systematic, disease-specific quantification of circadian disruption which so far has relied on annotating disease phenotypes separately by domain experts^45^. These results motivate and inform follow-up studies on whether maintaining or re-establishing strong, high amplitude temperature rhythms confer protection against developing chronic diseases. Clinical studies could, for example, deploy high heat capacity mattresses^46,47^ as an intervention to test whether this strategy could strengthen robustness of temperature biorhythms, and to discern underlying changes in the molecular fingerprint. Novel wearable omics devices that confer low patient burden can complement this to generate mechanistic insight^48^. These are natural steps to disentangle causal relationship where circadian disruption raises disease susceptibility and severity while many diseases disrupt circadian rhythms^26^. These insights are expected to empower a more nuanced personalization of circadian health^49^, balancing lifestyle risk factors and disease pathology.

Thermoregulation in humans enables body temperature to stay within a narrow, tightly controlled range where physiological processes run most efficiently. Core and peripheral body temperature are inversely coupled to regulate body temperature by balancing heat production and loss^10,11^. Deviation from this is controlled during a febrile response to fight pathogens, or uncontrolled in cases of prolonged extrinsically caused hyper- or hypothermia, often resulting in multi-organ failure. The circadian aspect of thermoregulation is achieved through diurnal oscillations of the temperature set point in the hypothalamic preoptic area and controlled by the ‘master’ clock in the suprachiasmatic nucleus (SCN). Thermogenic and heat-dissipative processes modulate the set point throughout the course of 24 hours typically resulting in peak core temperatures in the afternoon with the nadir reached at the end of the sleeping phase. These oscillatory temperature cues are likely picked up in the periphery to entrain, for example, sets of hepatic gene transcripts independent of peripheral liver clocks. This was suggested in a mouse model where local oscillators present in hepatocytes were silenced by means of a doxycycline-dependent REV-ERBa-mediated suppression of Bmal1^50^. Compared to controls, a set of 31 transcripts, among them core clock genes (Per2), members of the heat shock protein family and cold-inducible RNA-binding protein (Cirbp), continued to oscillate, supporting the hypothesis that entrainment is mediated through the SCN-temperature axis rather than by peripheral oscillators. For Cirbp, this was mechanistically further substantiated *ex vivo* in mouse broblasts which, when exposed to simulated body temperature cycles, started to oscillate^51^. RNA interference of Cirbp in these experiments demonstrated its role to drive circadian gene expression at high amplitudes. This robustness is driven by increasing rhythmic abundance of circadian clock gene transcripts, Clock and likely Rora, Ncor1, Sirt1, and Per3, in the cytoplasm^51^ through enhanced splicing efficiency as the likely post-transcriptional process^52^. This is consistent with Ki *et al*.^53^ who argue that the most thermo-sensitive mechanism is afforded by conformational changes of the RNA secondary structure. The internal ribosome entry site (IRES) is thought to be particularly prone to these temperature-divergent structural changes. IRES is responsible for mounting the cellular stress response under conditions of apoptosis, hypoxia, mitosis and nutrient deprivation through a cap-independent translational process, an alternative mechanism for protein production. IRES elements are found in about 10% of human 5’UTRs^54^. Dysregulation of this IRES-mediated translation in distinct transcripts, such as insulin-like growth factor 1 (IGF-1) receptor, are implicated under certain conditions with the corresponding pathology, i.e., diabetes (as suggested in Marques *et al*.^55^), pointing towards the clinical relevance of this pathway. In a cell culture model, IRES-specific translation was instrumental for murine Per1 oscillations^56^, a core regulator of the clock’s transcriptional feedback loop. This raises the question of whether a hampered IRES-dependent cellular stress response weakens the clock.

Of course, other mechanisms are at play. Temperature-dependent polyadenylation through RBM3, a cold-inducible RNA-binding protein, has been suggested to contribute to cell reprogramming during a stress response^53,57^. An explanation for some of the strong associations observed in this study might be that small amplitude temperature rhythms diminish thermosensitive gene regulation, which in combination with disease-specific perturbations, like those observed for diabetic neuropathy,^31,32^ lead to the emergence of specific disease phenotypes. We suggest that comprehensive studies, such as the one piloted in the human chronobiome^43^, are necessary to untangle mechanistic relationships. Time-integrated transomic assessments seem to be necessary to tease out how thermoregulatory rhythms entrained, for example, by microbiota^58^ fit into the picture.

We found a strong association between dampened temperature rhythms and mortality. The mortality rate was increased by 14% in those who displayed a wrist temperature amplitude that was diminished by two standard deviations (1.8°C); and the literature supports this association. For example, lifespan was increased in transgenic mice by 12% in males and 20% in females with strengthened temperature amplitudes^59^. And overexpression of uncoupling protein 2 in hypocretin neurons (Hcrt-UCP2) of these animals reduced core body temperature by 0.3–0.5°C accompanied (though this was not measured) by a presumable increase in peripheral temperature through the coupling of core and periphery in the thermoregulatory sleep model^10^.

One limitation of this study is that the wrist temperature readings were collected from a sensor enclosed within the wrist-worn actigraphy device where the sensor is separated from direct skin contact by a few millimeters of plastic. Our results indicate that the temperature traces in the UKBB study capture biological signal compellingly and agree with field studies^13,22,31^. Further support comes from a study where the skin temperature measurements did not correlate with ambient temperature readings from a second device placed nearby on the participants’ external clothing, suggesting that a device worn on the wrist sufficiently captures the biological signal and is little contaminated by ambient temperature^31^.

Nevertheless, ambient temperature fluctuations should be considered as a potential factor. Here, wearables with discrete temperature tracing^60^ offer complementary monitoring of thermoregulation in future efforts. A second limitation is that disease phenotypes are collected from in-patient hospital diagnostic codes. Therefore, some phenotypes may be recorded significantly later than actual disease onset and others may be missed from the dataset entirely. This was partially corrected for by excluding subjects according to prior diagnoses of any related disease, derived from including self-reported medical conditions at the initial assessment. Another limitation is that temperature rhythms are confounded by BMI as illustrated in Figure S2. Although we included BMI as a covariate in the models, its measurement preceded actigraphy by about five years, so it served as an incomplete control.

In conclusion, we established that decreased wrist temperature amplitudes predict future onset of diseases in participants of the UKBB, suggesting peripheral thermoregulation as a digital biomarker.

## Online Methods

### Actigraphy in the UK Biobank

The UKBB collected 7 days of actigraphy from 103,688 participants (Fig. 1)^19^ which were calibrated, processed^19,61–63^, and modified to report temperature values from an on-device temperature sensor. A total of 92,325 participants passed quality control (see Supplemental Methods) of which 91,462 had no missing data among the selected covariates and thus were selected for analysis. The overall correlation between physical activity and wrist temperature was weak (Spearman r = 0.21), indicating that temperature is a distinct measure despite being recorded from the same device as motion.

In addition, repeated measurements across seasons were collected for 3,197 participants which is available but has not yet been reported in detail. We leveraged these repeated datasets to account for seasonality across participants and to determine the variance of temperature amplitudes.

### Rhythm robustness

Diurnal rhythms were computed via cosinor fits^64^ of the wrist temperatures (Figure S1) in each participant, and amplitude parameter were extracted from each fit. These amplitudes were measured in degrees Celsius and give the difference between mesor (mid-line) and peak values of the t. Peak-to-trough values reflect twice the amplitude. Cosinor fits have been validated for use in measuring rhythms in both distal skin temperature and core body temperature^14,65^.

Since temperature is expected to be a highly seasonally variable, we corrected for season via a cosinor fit over time-of-year on the log temperature amplitude.

### Phenotypes

Diagnosis events were assessed in subjects starting one year after actigraphy, for a mean follow-up time of 5.9 years (4.8–7.3 min-max), see Fig. 1a. Events were identified from inpatient hospital record ICD-10 codes, which were grouped into phenotypes (PheCODEs)^24,25^ using the PheCODE Map v1.2b1. The PheCODE Map defines exclusion criteria for each phenotype, and subjects meeting those criteria (from ICD-10 and ICD-9 codes, or self-report) prior to the start of assessment (i.e., one year after actigraphy) were excluded from the analysis of that phenotype (Table S2, see Supplemental Methods 451,994 distinct patient-diagnoses (mean 4.9 per participant) and 3,061 deaths (3.3%) were recorded among these participants at the time of data download on 2/23/2022 (Fig. 1a).

### Proportional hazards models

A Cox proportional hazards model was performed for each PheCODE with ≥ 200 cases, based on a power analysis performed via simulation to establish a 0.2 log (hazard ratio) effect size at 80% power. The endpoint was diagnosis with the PheCODE, censored by death or end of data collection. The timescale utilized was years since actigraphy measurement. The predictor (independent) variable was temperature amplitude. The model included covariates for sex (male/female), ethnicity (white/other, since 97% are white^8^), smoking history (Prefer not to answer/Never/Previous/Current)), age at the time of actigraphy measure (40–55/55–60/60–65/65–70/70–80), BMI at assessment (continuous), college education (yes/no), Townsend deprivation index (continuous), alcohol consumption (Prefer not to answer/Daily or almost daily/Three or four times a week/Once or twice a week/One to three times a month/Special occasions only/Never)) and self-reported overall health (Do not know/Prefer not to answer/Excellent/Good/Fair/Poor), determined at initial assessment. Survival analysis was performed with the same model but with death as endpoint. A complete-case analysis was performed on 91,462 subjects with complete data (Fig. 1b). All covariates were assessed at initial enrollment in the UKBB, which had a mean of 5.7 (2.8–8.7 min-max) years before actigraphy collection. Results were computed in terms of the hazard ratio (HR) and its robust standard errors, performed by the R package *survival*^66^. In text, we report HR per 2 SD of amplitude, corresponding to 1.8°C; 16% of individuals have 1 SD below mean amplitude and 1.1% of the population have 2 SD below mean amplitude.

To check for sex-specific and age-dependent effects, further models were generated including interaction terms for sex and age at the time of actigraphy measurement. Results were checked for validity of proportional hazards assumption, non-linear effects, and competing outcomes, see Supplemental Methods.

## Figures and Tables

**Table 1:** Hazard Ratios for select diagnoses

PheCODE	HR at 2SD	HR at 1°C	N
NAFLD (“Other chronic nonalcoholic liver disease”)	1.91 (1.58–2.31)	1.43 (1.28–1.58)	603
Type 2 diabetes	1.69 (1.53–1.88)	1.34 (1.26–1.42)	1936
Extrapyramidal disease and abnormal movement disorders	1.67 (1.32–2.11)	1.33 (1.16–1.51)	293
Sleep disorders	1.50 (1.3–1.74)	1.25 (1.15–1.36)	864
Renal failure	1.25 (1.14–1.37)	1.13 (1.07–1.19)	2310
Essential hypertension	1.23 (1.17–1.3)	1.12 (1.09–1.16)	6143
Diverticulosis	1.20 (1.13–1.28)	1.11 (1.07–1.14)	4528
Pneumonia	1.22 (1.11–1.33)	1.11 (1.06–1.17)	2256
Disorders of lipoid metabolism	1.16 (1.09–1.24)	1.09 (1.05–1.13)	4072
Osteoarthrosis	1.12 (1.04–1.21)	1.06 (1.02–1.11)	2921
Anxiety disorders	1.11 (0.99–1.24)	1.06 (1.0–1.12)	1555
Parkinson’s disease	0.76 (0.59–0.97)	0.86 (0.75–0.98)	295

From the Cox proportional hazards models, wrist temperature amplitudes were predictive of disease outcomes. Twelve of the largest significant effect sizes are shown, as hazard ratios comparing mean to two standard deviations (SD) below, corresponding to 1.8°C, below mean amplitude, or comparing to 1°C below the mean, along with the number of events (cases). See also Table S1 for full results.
